# Solutions of nonlinear real world problems by a new analytical technique

**DOI:** 10.1016/j.heliyon.2018.e00913

**Published:** 2018-11-05

**Authors:** Liaqat Ali, Saeed Islam, Taza Gul, Muhammad Altaf Khan, Ebenezer Bonyah

**Affiliations:** a Department of Basic Sciences and Humanities, CECOS University Peshawer, KPK, Pakistan; b Institute of Management Sciences (IMSciences), KPK, Peshawar, Pakistan; c Department of Mathematics, Abdul Wali Khan University Mardan, KPK, Pakistan; d Department of Mathematics, City University of Science and Information Technology, Peshawar 25000, Khyber Pakhtunkhwa, Pakistan; e Department of Mathematics Education, University of Education – Winneba, Kumasi, Ghana

**Keywords:** Computational mathematics

## Abstract

Here a new analytical scheme is presented to solve nonlinear boundary value problems (BVPs) of higher order occurring in nonlinear phenomena. This method is called second alternative of Optimal Homotopy Asymptotic Method. It converts a complex nonlinear problem into zeroth order and first order problem. A homotopy and auxiliary functions which are consisted of unknown convergence controlling parameters are used in this technique. The unknown parameters are determined by minimizing the residual. Many methods are used to determine these parameters. Here Galerkin's method is used for this purpose. It is applied to solve non-linear BVPs of order four, five, six, and seven. The Consequences are compared with other methods e.g., Differential Transform Method (DTM), Adomain Decomposition Method (ADM), Variational Iteration Method (VIM), and Optimal Homotopy Asymptotic Method (OHAM). It gives efficient and accurate first-order approximate solution. The achieved results are compared with the exact solutions as well as with other methods to authenticate the applied technique. This method is very simple and easy but more operative.

## 1 Introduction

Many real world problems can be expressed in terms of BVPs. They have a significant contribution in almost every field. Many fluid problems can be modeled in terms of BVPs with various orders [1], [2], [3], [4]. Several efforts have been made to solve BVPs occurring in science and technology. Some of the numerical methods like finite element method, shooting method, and Runge Kutta method are helpful and have been used for the solution of such problems [5], [6], [7], [8]. Other approximate techniques like ADM and VIM are also helpful to use for this purpose [9], [10], [11]. Asymptotic and perturbation schemes are also used to gain the solutions of BVPs but unluckily these techniques are suitable for weak nonlinear problems and in general they dependent upon physical parameters. Therefore, many researchers tried to create some schemes which are not reliant on physical parameters at all and usable for nonlinear problems strongly. After great struggle Liao et al. introduce a scheme naming Homotopy Analysis Method (HAM) for the first time which independent of physical parameters [12], [13]. Various Investigators have created some other techniques based on Homotopy are HPM [10], [14], [15], [16], OHAM [17], [18], [19], [20], [21], [22], [23], [24], [25], [26], and Optimal Homotopy Perturbation Method (OHPM) [27], [28], [29], [30] to achieve the solution of BVPs. The new relevant work can also be seen in [31], [32], [33], [34], [35], [36], [37], [38], [39], [40], [41], [42], [43], [44], [45], [46]. Some work about the applied method can be seen in [47], [48], [49], [50], [51], [52], [53]. Inspired and aggravated by the continuing research in this area, we apply a new form of OHAM (OHAM-2) explained below for solving the nonlinear BVPs of order four, five, six, and seven as given in [11], [25], [26], [54], [55], [56].

## 2 Methodology

### 2.1 Basic idea of OHAM

Consider the following BVP:

(1) $$\zeta (\omega (s))+g(s)+\varphi (\omega (s))=0,\;B\left(\omega (s),\;\frac{d\omega (s)}{ds}\right)=0.$$

Here, ω(s) is an undetermined, *ζ*, *φ* are linear and nonlinear operators, g(s) is a given function. Create a homotopy as: $ℑ(v(s,\sigma ,c_{i})):℘\times [0,1]\to ℜ$ so that

(2) $$\begin{array}{c} \;ℑ(v(s,\sigma ,c_{i}))=(1-\sigma )(\zeta (v(s,\sigma ,c_{i})+g(s))\\ =ℑ(s,\sigma ,c_{i})(\zeta (v(s,\sigma ,c_{i})+g(s)+\varphi (v(s,\sigma ,c_{i})). \end{array}$$

Where s∈ℜ, ℘ is the domain of interest. $ℑ(s,\sigma ,c_{i})$ presents auxiliary function.

Let the solution of Eq. (2) be in form given below:

(3) $$v(s,\sigma ,c_{i})=\omega_{0}(s)+\sum_{i\geq 1}\omega_{i}(s,c_{i})\sigma^{j},j=1,2,\ldots s.$$

Put σ=0 and σ=1, in Eq. (3) to get Eq. (4) and Eq. (5) as:

(4) $$v(s,0,c_{i})\;=\;\omega_{0}(s).$$

(5) $$v(s,1,c_{i})\;=\;\omega (s,c_{i}).$$

So, the solution $v(s,\sigma ,c_{i})$ converts from $\omega_{0}(s)$ to $\omega (s,c_{i})$ as *σ* vary from 0 to 1. Substitute Eq. (3) in Eq. (2) and compare coefficients of *σ* on both sides to get Eq. (6) to Eq. (8) given below:

Zero order problem:

(6) $$\zeta (\omega_{0}(s))\;+g(s)\;=0\;,\;B(\omega_{0}(s),\;\frac{d\omega_{0}(s)}{ds})\;=\;0.$$

First order problem:

(7) $$\zeta (\omega_{1}(s))=c_{1}\varphi_{0}(\omega_{0}(s)),\;B(\omega_{1}(s),\frac{d\omega_{1}(s)}{ds})=0.$$

For $\omega_{k}(s)$:

(8) $$\begin{array}{c} \zeta (\omega_{k}(s))-\zeta (\omega_{k-1}(s))\;=c_{k}\;\varphi_{0}(\omega_{0}(s))+\\ \sum_{i=1}^{k-1}c_{i}[\zeta (\omega_{k-i}(s))+\varphi_{k-i}(\omega_{0}(s),\omega_{1}(s),...,\omega_{k-1}(s))],\\ k=2,3,...,B(\omega_{k},\frac{du_{k}}{ds})=0, \end{array}$$

where $\varphi_{m}(\omega_{0}(s),\omega_{1}(s),\omega_{2}(s),\ldots ,\omega_{m}(s))$ is the coefficient of $\sigma^{m}$ when $\varphi (v(s,\sigma ,c_{i}))$ expand about the embedding parameter *σ*: to get Eq. (9) as:

(9) $$\begin{array}{ccc} \varphi (v(s,\sigma ,c_{i}) & = & \varphi (\omega_{0}(s))+\varphi (\omega_{0}(s),(\omega_{1}(s),c_{j}))\sigma \\ & & +\varphi (\omega_{0}(s),(\omega_{1}(s),c_{j}),(\omega_{2}(s),c_{j}))\sigma^{2}+\ldots . \end{array}$$

If σ=1, then Eq. (3) is convergent and changes to the form of Eq. (10) which is convergent:

(10) $$v(s,1,c_{i})=\omega (s,c_{i})=v(s,\sigma ,c_{i})=\omega_{0}(s)+\sum_{i\geq 1}\omega_{i}(s,c_{i}),j=1,2,\ldots s.$$

Therefore the *m*th-order approximate solution of Eq. (1) is denoted by Eq. (11):

(11) $$\omega (s,c_{j})=\overset{˜}{\omega }(s,c_{j})=\omega_{0}(s)+\sum_{i=1}^{m}\omega_{i}(s,c_{j}),j=1,2,3,\ldots s.$$

Put Eq. (11) in Eq. (1) to gain the residual shown by Eq. (12):

(12) $$ℜ(s,c_{1},c_{2},\ldots ,c_{m})=\zeta (\overset{˜}{\omega }(s,c_{j})+\varphi (\overset{˜}{\omega }(s,c_{j})+g(s).$$

For $c_{i},i=1,2,\ldots$ and to minimize the residuals use Eq. (13) to Eq. (14)

(13) $$\zeta (c_{1},c_{2},\ldots ,c_{m})=\int_{a}^{b}{ℜ}^{2}(s,c_{1},c_{2},\;\ldots ,c_{m})ds,$$

and

(14) $$\frac{\partial \zeta }{\partial c_{1}}=\frac{\partial \zeta }{\partial c_{2}}=\;\ldots =\frac{\partial \zeta }{\partial c_{m}}=0,$$

or Eq. (15)

(15) $$\int_{a}^{b}ℜ\frac{\partial \overset{˜}{\omega }}{\partial c_{1}}ds=0,\;\int_{a}^{b}ℜ\frac{\partial \overset{˜}{\omega }}{\partial c_{2}}ds=0,\ldots .$$

### 2.2 Second alternative of OHAM (OHAM-2) [20]

Use the basic idea of OHAM to achieve OHAM-2 mentioned in the book [17] and [47].

Consider Eq. (1) and take $v(s,\sigma ,c_{i})$ in a particular form indicated in Eq. (16):

(16) $$v(s,\sigma ,c_{i})=\omega_{0}(s)+\sigma \;\omega_{1}(s,c_{i}),$$

put the above value in Eq. (2) to achieve Eq. (17) as below:

(17) $$\begin{array}{c} ℑ[\zeta (v(s,\sigma ,c_{i}))+g(s),\;ℑ(s,c_{i}),\;\varphi (v(s;\sigma ,c_{i})]\\ =\zeta (\omega_{0}(s))+g(s)+\sigma [\zeta (\omega_{1}(s,c_{i}))-ℑ(s,c_{i})\varphi (\omega_{0}(s))], \end{array}$$

which fulfill the characteristics:

(18) $$ℑ[\zeta (v(s,0,c_{i}))+g(s),ℑ(s,c_{i}),\;\varphi (v(s;0,c_{i})]=\zeta (\omega_{0}(s))+g(s)=0,$$

(19) $$\begin{array}{c} ℑ[\zeta (v(s,1,c_{i}))+g(s),ℑ(s,c_{i}),\varphi (v(s;1,c_{i})]\\ =\zeta (\omega_{1}(s,c_{i}))-ℑ(s,c_{i})\varphi (\omega_{0}(s))=0, \end{array}$$

$ℑ(s,c_{i})\neq 0$ is an auxi. ftn and the terms in $\sigma^{2}$ are omitted.

$\omega_{0}(s)$ is calculated from Eq. (18) as:

(20) $$\zeta (\omega_{0}(s))+g(s)=0,\;B\left(\omega_{0}(s),\;\frac{d\omega_{0}(s)}{ds}\right)=0.$$

$\omega_{1}(s,c_{i})$ is can be obtained from Eq. (19) given below: 1st order approximation $\omega_{1}(s,c_{i})$, i.e.

(21) $$\begin{array}{c} \zeta (\omega_{1}(s,c_{i}))=ℑ(s,c_{i})\varphi (\omega_{0}(s)),\;B\left[\omega_{1}(s,c_{i}),\;\frac{d\omega_{1}(s,c_{i})}{ds}\right]=0,\\ i=1,\;2,\;\ldots ,\;s. \end{array}$$

Use Eq. (20) and Eq. (21) to gain the 1st-order approximate solution shown in Eq. (22):

(22) $$\overset{˜}{\omega }(s,c_{i})=\omega (s,c_{i})=\omega_{0}(s)+\;\omega_{1}(s,c_{i}),$$

$c_{1},c_{2},\ldots ,c_{s}$ are auxiliary parameters.

### 2.3 Applications of method (OHAM-2)

Here the proposed technique is applied to some non linear BVPs of different orders and high accuracy of OHAM-2 is illustrated. The results are also compared with other methods to authenticate the code.

### 2.4 Model 1. Consider non-linear BVP of order four as solved by Murad et al. in [54]

(23) $$\frac{d^{4}\omega (s)}{ds^{4}}+2\frac{\rho }{\omega }\;\omega (s)\;\frac{d^{3}\omega (s)}{ds^{3}},\;0\leq \;s\;\leq 1,$$

with BCs,

(24) $$\omega (0)=0,\;\omega^{\prime \prime }(0)=0,\;\omega (1)=1,\;\omega^{\prime }(1)=\gamma \;\omega^{\prime \prime }(1).$$

OHAM-2 is applied as,

Zeroth order problem:

(25) $$\begin{array}{c} \omega_{0}^{⁗}(s)=0,\\ \omega_{0}(0)=0,\;\omega_{0}^{\prime \prime }(0)=0,\;\omega_{1}(1)=0,\;\omega_{0}^{\prime }(1)=\gamma \;\omega^{\prime \prime }(1), \end{array}$$

which gives,

(26) $$\omega_{0}(s)=\frac{1}{4}\left(3s+s^{3}\right)$$

Now, since $\varphi (\omega (s))=ℜ\omega (s)\;\omega^{\prime \prime \prime }(s)$ which gives $\varphi (\omega_{0}(s))=\frac{3}{8}\left(3s+s^{3}\right)$ when $ℜ=2\frac{\rho }{\omega }=1$ [54]. Therefore according to the above method mentioned, choose the auxi. ftn as:

(27) $$ℑ(s,c_{i})={ℑ}_{1}(s,{ℑ}_{1},c_{i})=c_{1}+c_{2}s+c_{3}s^{2}+c_{4}s^{3}.$$

Use Eq. (25), Eq. (26) and Eq. (27) in Eq. (21) to get Eq. (28):

1st order problem:

(28) $$\begin{array}{c} \omega_{1}^{⁗}(s,c_{i})=(c_{1}+c_{2}s+c_{3}s^{2}+c_{4}s^{3})\varphi (\omega_{0}(s)),\\ \omega_{1}(0)=0,\;\omega_{1}^{\prime \prime }(0)=0,\;\omega_{1}(1)=0,\;\omega_{1}^{\prime }(1)=\gamma \;\omega_{1}^{\prime \prime }(1). \end{array}$$

Use Galerkin's technique to achieve the following values mentioned in Eq. (29):

(29) $$c_{1}=-1.11431,\;c_{2}=-0.0272491,\;c_{3}=0.373307,\;c_{4}=0.0118121,$$

put the above values of $\omega_{0}(s)$, $\omega_{1}(s,c_{i})$, γ=1, and σ=1 in Eq. (22) to achieve the solution of Eq. (23) and Eq. (24) as represented in Eq. (30):

(30) $$\begin{array}{c} \omega (s)=0.718965s+0.291515s^{3}-0.0104466s^{5}-0.0000851535s^{6}+\\ 2.506581953200744\times 10^{-6}s^{7}+1.82751995620187\times 10^{-6}s^{8}+\\ 0.0000462931s^{9}+8.788790842057447\times 10^{-7}s^{10}. \end{array}$$

**Figure 1:**
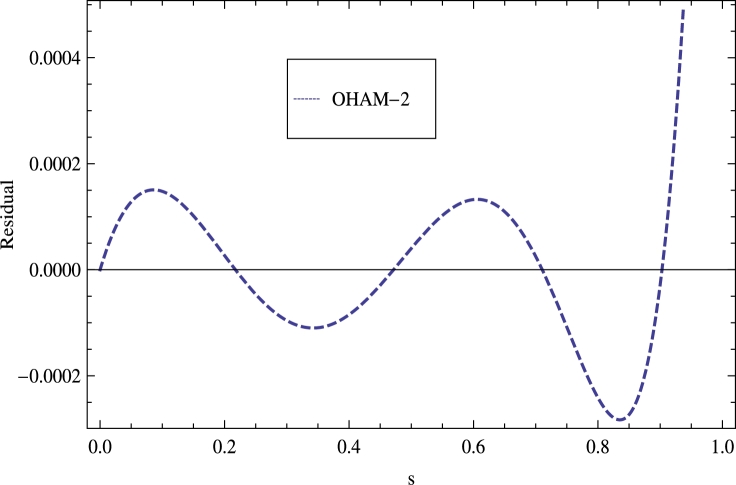
Shows residuals graph of the solution achieved by OHAM-2.

The results are illustrated in the Table 1 and Figure 1 for model 1.

**Table 1:** Indicates comparison of the errors gained by method OHAM in [54] and OHAM-2 for model 1, *E*^⁎^ = Exact-Numerical.

s	Numerical	OHAM	OHAM-2	$E^{⁎}$ (OHAM)	$E^{⁎}$ (OHAM-2)	Residual (OHAM-2)
0.0	0.	0.	0.	0.	0.0	0.0
0.1	0.0721879	0.0721919	0.0721879	−4.0 × 10^−6^	−9.8 × 10^−9^	1.5 × 10^−4^
0.2	0.146122	0.146129	0.146122	−7.0 × 10^−6^	2.3 × 10^−7^	2.7 × 10^−5^
0.3	0.223535	0.223544	0.223535	−9.0 × 10^−6^	3.6 × 10^−8^	−9.6 × 10^−5^
0.4	0.306136	0.306146	0.306136	−1.0 × 10^−5^	3.3 × 10^−7^	4.0 × 10^−5^
0.5	0.395594	0.395603	0.395594	−9.0 × 10^−6^	−2.4 × 10^−7^	8.5 × 10^−5^
0.6	0.493531	0.493538	0.493531	−7.0 × 10^−6^	4.3 × 10^−7^	1.3 × 10^−4^
0.7	0.601502	0.601507	0.601502	−5.0 × 10^−6^	3.4 × 10^−7^	2.5 × 10^−5^
0.8	0.720989	0.720993	0.720989	−4.0 × 10^−6^	−4.8 × 10^−7^	−2.4 × 10^−4^
0.9	0.853389	0.853391	0.853389	−2.0 × 10^−6^	−4.8 × 10^−7^	−2.8 × 10^−5^
1.	1.	1.	1.	0.	0.	2.5 × 10^−3^

### 2.5 Model 2. Consider non-linear BVP of order five [25]

(31) $$\frac{d^{5}\omega (s)}{ds^{5}}=\;1/32\omega^{3}(s)\;e^{-s}\;,\;0\;\leq \;s\;\leq \;1,$$

with BCs,

(32) $$\omega (0)=1,\;\omega^{\prime }(0)=1/2,\;\omega^{\prime \prime }(0)=1/4,\;\omega (1)=e^{1/2},\;\omega^{\prime }(1)=1/2e^{1/2},$$

and exact solution $\omega (s)=e^{s/2}$.

Now we use OHAM-2:

$\omega_{0}(s)$ can be calculated as

Zeroth order problem:

(33) $$\begin{array}{c} \omega_{0}^{\prime \prime \prime \prime \prime }(s)=0,\;\omega_{0}(0)=1,\;\omega_{0}^{\prime }(0)=1/2,\\ \omega_{0}^{\prime \prime }(0)=1/4,\;\omega_{0}(1)=e^{1/2},\;\omega_{0}^{\prime }(1)=1/2e^{1/2}, \end{array}$$

which gives

(34) $$\omega_{0}(s)=1.+0.5s+0.125s^{2}+0.0205244s^{3}+0.00319682s^{4}.$$

Since, $\varphi (\omega (s))=-1/32\omega^{3}e^{-s}$, therefore $\varphi (\omega_{0}(s))=\omega_{0}^{3}(s)e^{-s}$. Take

(35) $$ℑ(s,c_{i})=c_{1}+c_{2}s+c_{3}s^{2}+c_{4}s^{3}+c_{5}s^{4}.$$

1st order problem:

(36) $$\begin{array}{c} \omega_{1}^{\prime \prime \prime \prime \prime }(s,c_{i})=\left(c_{1}+c_{2}s+c_{3}s^{2}+c_{4}s^{3}+c_{5}s^{4}\right)\varphi (\omega_{0}(s)),\\ \omega_{1}(0)=0,\;\omega_{1}^{\prime }(0)=0,\;\omega_{1}^{\prime \prime }(0)=0,\;\omega_{1}(1)=0,\;\omega_{1}^{\prime }(1)=0. \end{array}$$

Use Galerkin's method to obtain the following values,

(37) $$\begin{array}{c} c_{1}=1.00001,\;c_{2}=-0.000186167,\;c_{3}=0.000942618,\;c_{4}=-0.00134276,\\ \;c_{5}=0.00057069. \end{array}$$

Put the values achieved from Eq. (33) to Eq. (37) in Eq. (22) to obtain the solution of Eq. (31) and Eq. (32) shown in Eq. (38) given below,

(38) $$\begin{array}{c} \omega (s)=1.+0.5s+0.125s^{2}+0.0208333s^{3}+0.00260417s^{4}+0.00026042s^{5}\\ +0.0000216936s^{6}+1.560654622491429\times 10^{-6}s^{7}\\ +8.841246644373896\times 10^{-8}s^{8}+9.084874135580505\times 10^{-9}s^{9}\\ -3.98263569776148\times 10^{-10}s^{10}+3.2224483073578952\times 10^{-12}s^{11}\\ +2.1428840357653123\times 10^{-13}s^{12}+1.0295165538329375\times 10^{-12}s^{13}\\ -2.669017709456431\times 10^{-13}s^{14}. \end{array}$$

**Figure 2:**
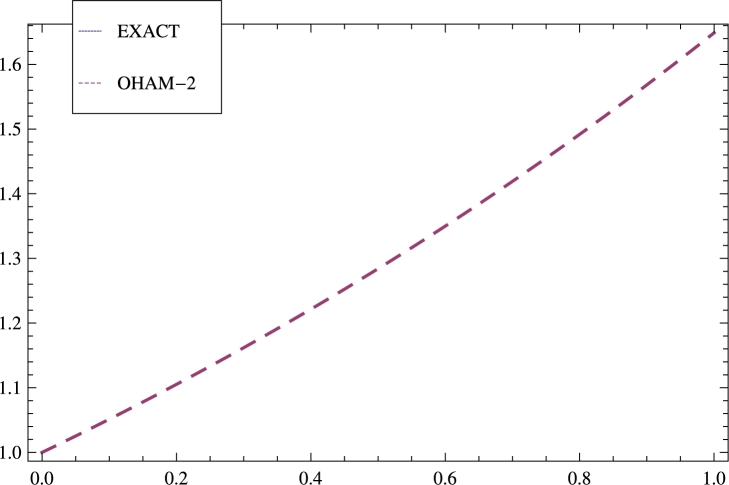
The results obtained by OHAM-2 are compared with the exact solution as well as with [25] for model 2.

The results are shown in Table 2 and Figure 2 for model 2.

**Table 2:** Illustrates comparison of the errors gained by methods: DTM in [56], OHAM in [25] and OHAM-2.

s	Exact	OHAM-2	$E^{⁎}$ (DTM)	$E^{⁎}$ (OHAM)	$E^{⁎}$ (OHAM-2)
0.0	1.0	1.	0.0000	0.0000	0.0000
0.1	1.05127	1.05127	1.0 × 10^−9^	−9.2 × 10^−10^	−4.4 × 10^−14^
0.2	1.10517	1.10517	2.0 × 10^−9^	−5.0 × 10^−9^	−1.5 × 10^−13^
0.3	1.16183	1.16183	1.0 × 10^−8^	−1.1 × 10^−8^	−2.5 × 10^−13^
0.4	1.2214	1.2214	2.0 × 10^−8^	−1.5 × 10^−8^	−3.3 × 10^−13^
0.5	1.28403	1.28403	3.0 × 10^−8^	−1.6 × 10^−8^	−3.8 × 10^−13^
0.6	1.34986	1.34986	3.7 × 10^−8^	−1.4 × 10^−8^	−3.7 × 10^−13^
0.7	1.41907	1.41907	4.1 × 10^−8^	−9.9 × 10^−9^	−2.9 × 10^−13^
0.8	1.49182	1.49182	3.1 × 10^−8^	−5.6 × 10^−9^	−1.8 × 10^−13^
0.9	1.56831	1.56831	1.4 × 10^−8^	−1.1 × 10^−9^	−5.2 × 10^−14^
1.0	1.64872	1.64872	0.0000	0.0000	5.8 × 10^−14^

### 2.6 Model 3. Assume non-linear BVP of order six solved by Haziqah et al. [56]

(39) $$\frac{d^{6}\omega (s)}{ds^{6}}=1/64\;\omega^{3}(s)\;e^{s}\;,\;0\;\leq \;s\;\leq \;1.$$

The boundary conditions for this model are:

$$\begin{array}{cc} & \omega (0)=1,\omega^{\prime }(0)=-1/2,\;\omega^{\prime \prime }(0)=1/4,\\ & \omega (1)=e^{-1/2},\omega^{\prime }(1)=-1/2e^{-1/2},\omega^{\prime \prime }(1)=1/4e^{-1/2}, \end{array}$$

and the exact solution is $\omega (s)=e^{-s/2}$

To apply the method we first find the initial guess:

$\omega_{0}(s)$ be determined as

Zeroth order problem:

(40) $$\begin{array}{c} \omega_{0}^{\prime \prime \prime \prime \prime \prime }(s)=0,\;\omega_{0}(0)=1,\omega_{0}^{\prime }(0)=-1/2,\;\omega_{0}^{\prime \prime }(0)=1/4,\\ \omega_{0}(1)=e^{-1/2},\omega_{0}^{\prime }(1)=-1/2e^{-1/2},\omega_{0}^{\prime \prime }(1)=1/4e^{-1/2}, \end{array}$$

which gives

(41) $$\begin{array}{ccc} \omega_{0}(s) & = & -0.00020372(-4.88093+s)(25.8548-7.61713s+s^{2})\\ & & \times (38.8977-0.0197842s+s^{2}). \end{array}$$

Since, $\varphi (\omega (s))=-1/64\omega^{3}e^{s}$, therefore $\varphi (\omega_{0}(s))=\omega_{0}^{3}(s)e^{s}$. Take the auxiliary function as:

(42) $$ℑ(s,c_{i})=c_{1}+c_{2}\;s+c_{3}\;s^{2},$$

to achieve $\omega_{1}(s)$ from the following.

1st order problem:

(43) $$\begin{array}{c} \omega_{1}^{\prime \prime \prime \prime \prime \prime }(s,c_{i})=\left(c_{1}+c_{2}\;s+c_{3}\;s^{2}\right)\varphi (\omega_{0}(s)),\\ \omega_{1}(0)=0,\;\omega_{1}^{\prime }(0)=0,\;\omega_{1}^{\prime \prime }(0)=0,\omega_{1}(1)=0,\;\omega_{1}^{\prime }(1)=0,\;\omega_{1}^{\prime \prime }(1)=0. \end{array}$$

Use Galerkin's method to obtain the following:

(44) $$\begin{array}{c} c_{1}=1,\;c_{2}=-7.622858726619207\times 10^{-6},\\ c_{3}=7.425899889285021\times 10^{-6}. \end{array}$$

Put the values achieved from Eq. (40) to Eq. (44) in Eq. (22) to get the solution of Eq. (39) and Eq. (40) shown in Eq. (45) given below,

(45) $$\begin{array}{c} \omega =1.-0.5s+0.125s^{2}-0.0208333s^{3}+0.00260417s^{4}-0.000260417s^{5}\\ +0.0000217014s^{6}-1.550124367417937\times 10^{-6}s^{7}\\ +9.68900054277912\times 10^{-8}s^{8}\\ -5.369872423531197\times 10^{-9}s^{9}+2.5247466976003126\times 10^{-10}s^{10}\\ -4.250971361237948\times 10^{-12}s^{11}-1.0189100394276824\times 10^{-12}s^{12}\\ +3.9136162828262685\times 10^{-14}s^{13}-1.3159716659631081\times 10^{-15}s^{14}. \end{array}$$

**Figure 3:**
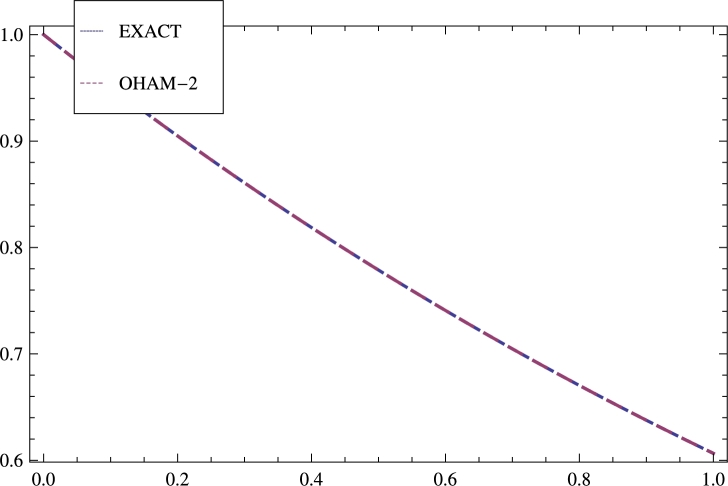
Illustrates comparison of the solution obtained by OHAM-2 with the exact solution as well as with the results achieved by Methods: DTM and ADM in [56] for model 3.

Table 3 and Figure 3 shows the results for model 3.

**Table 3:** The results obtained DTM, ADM in [56], and OHAM-2 are compared.

s	Exact	OHAM-2	$E^{⁎}$ (DTM) (N=18)	$E^{⁎}$ (ADM) (N=18)	$E^{⁎}$ (OHAM-2)
0.0	1.	1.	0.0000	0.0000	0.0000
0.1	0.951229	0.951229	2.0 × 10^−3^	2.0 × 10^−3^	1.1 × 10^−16^
0.2	0.904837	0.904837	1.3 × 10^−2^	1.3 × 10^−2^	−1.1 × 10^−16^
0.3	0.860708	0.860708	3.6 × 10^−2^	3.6 × 10^−2^	−1.1 × 10^−16^
0.4	0.818731	0.818731	6.5 × 10^−2^	6.5 × 10^−2^	−1.1 × 10^−16^
0.5	0.778801	0.778801	9.5 × 10^−2^	9.5 × 10^−2^	1.1 × 10^−16^
0.6	0.740818	0.740818	1.1 × 10^−1^	1.2 × 10^−1^	2.2 × 10^−16^
0.7	0.704688	0.704688	9.8 × 10^−6^	1.2 × 10^−1^	0.0000
0.8	0.67032	0.67032	4.3 × 10^−6^	9.9 × 10^−2^	0.0000
0.9	0.637628	0.637628	7.6 × 10^−7^	5.7 × 10^−2^	−1.1 × 10^−16^
1.0	0.606531	0.606531	−1.0 × 10^−10^	8.0 × 10^−10^	−2.2 × 10^−16^

### 2.7 Model 4. Consider non-linear BVP of order seven solved by Siddiqui et al. [11]

(46) $$\frac{d^{7}\omega (s)}{ds^{7}}=\;\omega^{2}(s)\;e^{-s}\;,\;0\;\leq \;s\;\leq \;1,$$

with BCs.:

(47) $$\begin{array}{c} \omega (0)=1,\;\omega^{\prime }(0)=1,\;\omega^{\prime \prime }(0)=1,\;\omega^{\prime \prime \prime }(0)=1,\\ \omega (1)=e,\;\omega^{\prime }(1)=e,\;\omega^{\prime \prime }(1)=e. \end{array}$$

The exact solution for this model is $\omega (s)=e^{s}$. Now we use the proposed method: The initial guess $\omega_{0}(s)$ is determined as

Zeroth order problem:

(48) $$\begin{array}{c} \omega_{0}^{\prime \prime \prime \prime \prime \prime \prime }(s)=0,\\ \omega_{0}(0)=1,\;\omega_{0}^{\prime }(0)=1,\;\omega_{0}^{\prime \prime }(0)=1,\;\omega_{0}^{\prime \prime \prime }(0)=1,\omega_{0}(1)=e,\;\omega_{0}^{\prime }(1)=e,\\ \omega_{0}^{\prime \prime }(1)=e, \end{array}$$

which gives

(49) $$\begin{array}{ccc} \omega_{0}(s) & = & 1.+1.s+0.5s^{2}+0.166667s^{3}+0.0419592s^{4}+0.00749074s^{5}\\ & & +0.00216522s^{6}. \end{array}$$

Since, $\varphi (\omega (s))=\omega^{2}e^{-s}$, therefore $\varphi (\omega_{0}(s))=\omega_{0}^{2}(s)e^{-s}$. Take auxiliary function as

(50) $$ℑ(s,c_{i})=c_{1}+c_{2}s+c_{3}s^{2}+c_{4}s^{3}+c_{5}s^{4},$$

and obtained $\omega_{1}(s)$ from the following equation

1st order problem:

(51) $$\begin{array}{c} \omega_{1}^{\prime \prime \prime \prime \prime \prime }(s,c_{i})=(c_{1}+c_{2}s+c_{3}s^{2}+c_{4}s^{3}+c_{5}s^{4})\varphi (\omega_{0}(s)),\\ \omega_{1}(0)=0,\;\omega_{1}^{\prime }(0)=0,\;\omega_{1}^{\prime \prime }(0)=0,\;\omega_{1}^{\prime \prime \prime }(0)=0,\\ \omega_{1}(1)=0,\;\omega_{1}^{\prime }(1)=0,\;\omega_{1}^{\prime \prime }(1)=0. \end{array}$$

Apply Galerkin's method to obtain the following values,

(52) $$\begin{array}{c} c_{1}=0.999995,\;c_{2}=0.0000667923,\;c_{3}=-0.000290255,\;c_{4}=0.000447228,\\ \;c_{5}=-0.000231963. \end{array}$$

Put the values achieved from Eq. (48) to Eq. (52) in Eq. (22) to get the solution of Eq. (46) and Eq. (47) inculcated in Eq. (53) given below.

So, solution becomes as

(53) $$\begin{array}{c} \omega =1.+1.s+0.5s^{2}+0.166667s^{3}+0.0416667s^{4}+0.00833333s^{5}\\ +0.00138889s^{6}+0.000198412s^{7}+0.0000248031s^{8}\\ +2.7544857434993286\times 10^{-6}s^{9}\\ +2.7588649849892085\times 10^{-7}s^{10}+2.5452607840240587\times 10^{-8}s^{11}\\ +1.651990742551977\times 10^{-9}s^{12}+3.339564525517191\times 10^{-10}s^{13}\\ -1.2713508780379934\times 10^{-11}s^{14}. \end{array}$$

**Figure 4:**
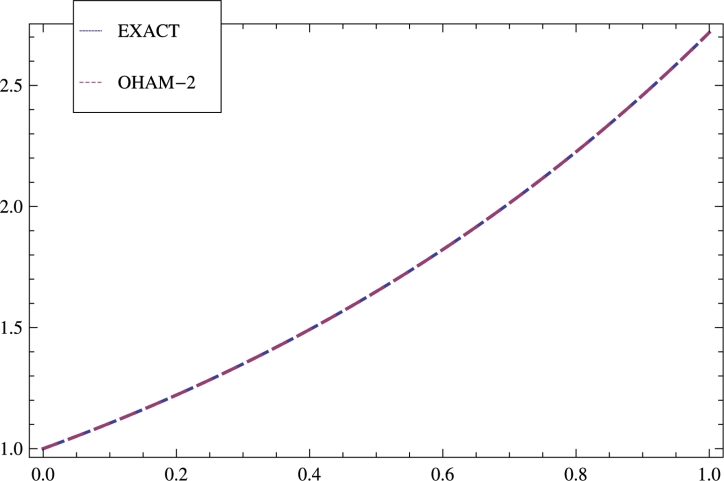
Shows comparison of the solution obtained by OHAM-2 with the exact solution a well as with the results achieved by VIM in [11] for model 4.

The outcomes are elaborated in the Table 4 and Figure 4 for model 4.

**Table 4:** VIM in [11], and OHAM-2.

s	Exact	OHAM-2	$E^{⁎}$ VIM	$E^{⁎}$ OHAM-2
0.0	1.	1.	0.0000	0.0000
0.1	1.10517	1.10517	3.8 × 10^−12^	−4.4 × 10^−15^
0.2	1.2214	1.2214	1.2 × 10^−10^	−5.1 × 10^−14^
0.3	1.34986	1.34986	2.8 × 10^−10^	−1.8 × 10^−13^
0.4	1.49182	1.49182	7.6 × 10^−10^	−4.0 × 10^−13^
0.5	1.64872	1.64872	1.2 × 10^−9^	−6.4 × 10^−13^
0.6	1.82212	1.82212	1.3 × 10^−9^	−7.7 × 10^−13^
0.7	2.01375	2.01375	1.2 × 10^−9^	−7.0 × 10^−13^
0.8	2.22554	2.22554	6.6 × 10^−10^	−4.4 × 10^−13^
0.9	2.4596	2.4596	1.7 × 10^−10^	−3.1 × 10^−13^
1.0	2.71828	2.71828	1.3 × 10^−11^	−1.1 × 10^−12^

## 3 Conclusions

Four nonlinear BVPs are solved by the new form of OHAM forming in different nonlinear phenomena. Auxiliary functions, Initial guess, Optimal convergence control parameters, Galerkin's method, Least square have great effect on the solution and increase the accuracy. The results gained by OHAM-2 are compared with the results achieved by VIM, OHAM, DTM, and ADM to inculcate the difference. This technique can solve nonlinear problems of any order easily. The results achieved by OHAM-2 are shown in tabular form as well as graphically. The errors gained by OHAM-2 are compared with the errors obtained Galerkin's Method with Quintic B-splines, and OHAM are shown in Table 1 for model 1. The outcomes are elaborated graphically in Figure 1, Figure 2, Figure 3 and Figure 4. Table 2 illustrates comparison of the errors gained by methods: DTM, OHAM, and OHAM-2 for model 2. Table 3 inculcates comparisons of the errors achieved by methods: DTM, ADM, and OHAM-2 for model 3 and finally comparisons of the errors achieved by methods: VIM and OHAM-2 are illustrated in Table 4 for model 4.

## Declarations

### Author contribution statement

Liaqat Ali: Conceived and designed the analysis; Analyzed and interpreted the data; Contributed analysis tools or data.

Saeed Islam, Muhammad A. Khan: Analyzed and interpreted the data.

Taza Gul: Conceived and designed the analysis.

Ebenezer Bonyah: Contributed analysis tools or data; Wrote the paper.

### Funding statement

The authors received no funding from an external source.

### Competing interest statement

The authors declare no conflict of interest.

### Additional information

No additional information is available for this paper.
